# A new species within the *Centaureabusambarensis* complex (Asteraceae, Cardueae) from Sicily

**DOI:** 10.3897/BDJ.10.e91505

**Published:** 2022-10-06

**Authors:** Gianniantonio Domina, Emilio Di Gristina, Giulio Barone

**Affiliations:** 1 Department of Agricultural, Food and Forest Sciences, University of Palermo, Palermo, Italy Department of Agricultural, Food and Forest Sciences, University of Palermo Palermo Italy; 2 Department of Architecture, University of Palermo, Palermo, Italy Department of Architecture, University of Palermo Palermo Italy

**Keywords:** *Centaurea*, endemism, Mediterranean area, mountain flora

## Abstract

**Background:**

The *Centaureabusambarensis* group is made up by eight species endemic to Sicily. We statistically evaluated a population found on the Nebrodi Mountain (NE Sicily) to verify if the observed morphological differences with the already known taxa justified the description of a new one. It resulted in being sufficiently distinct to deserve recognition at the species level.

**New information:**

*Centaureavaldemonensis*, a new species endemic to Sicily is described and illustrated here. It is confined to the Nebrodi Mountains (NE Sicily). The distinction of this taxon from the others belonging to the *C.busambarensis* complex has been supported with the aid of statistical analyses on morphological characters. The differences with the related taxa are discussed.

## Introduction

The *Centaureabusambarensis* group as delimited by Domina et al. (2017) is made up by eight species endemic to Sicily (central Mediterranean). It is included in what has been called the *C.cineraria* aggregate, a group of perennial herbs characterised by a white or grey indumentum, pinnatisect leaves, phyllaries with a decurrent scarious appendage and flexible fimbriae and pink tubular florets. According to Cela Renzoni and Viegi (1982), this aggregate includes species endemic to Italy (Pignatti and Lausi 1982, Bartolucci et al. 2018) and the N African *C.papposa* (Coss.) Greuter. The *C.cineraria* aggregate has been studied with the use of molecular techniques, by Hilpold et al. 2011 and Hilpold et al. 2014. They demonstrated that this aggregate is monophyletic with the exclusion of three species (*C.gymnocarpa* Moris & De Not., *C.veneris* (Sommier) Béeg. and *C.leucadea* Lacaita) and the inclusion of the little morphologically related Sicilian *C.parlatoris* Heldr.

The nomenclature and taxonomy of the *C.busambarensis* group and its related *C.parlatoris* complex have been under study for several years (e.g. Raimondo and Bancheva 2004, Raimondo et al. 2004, Palla et al. 2005, Bancheva et al. 2006 and Geraci et al. 2007) Bancheva et al. 2006, Geraci et al. 2007, Palla et al. 2005, Raimondo and Bancheva 2004, Raimondo et al. 2004) and recently revised by Domina et al. (2016), Domina et al. (2017)and Domina et al. (2021). They are polymorphic in several characters, which explains the high number of taxa described. However, intra-populational variation is considerable, even at the classical localities of the described taxa. It has been, therefore, difficult to draw clear-cut limits between these taxa.

The *C.busambarensis* complex treated here consists of: *C.augusae* Domina, Greuter & Raimondo, *C.busambarensis* Guss, *C.erycina* Raimondo & Bancheva, *C.panormitana* Lojac., *C.saccensis* Raimondo, Bancheva & Ilardi, *C.seguenzae* (Lacaita) Brullo, Marceno & Siracusa, *C.thyrrena* C.Brullo, Brullo & Giusso and *C.todaroi* Lacaita.

In the course of floristic investigations, a population apparently similar to *C.busambarensis*, but with evident morphological differences, was identified on the Rocche del Crasto, Nebrodi Mountains (NE Sicily) at high altitudes. We statistically evaluated whether these differences were such as to justify the description of a new taxon.

## Materials and methods

**Figure 1.**
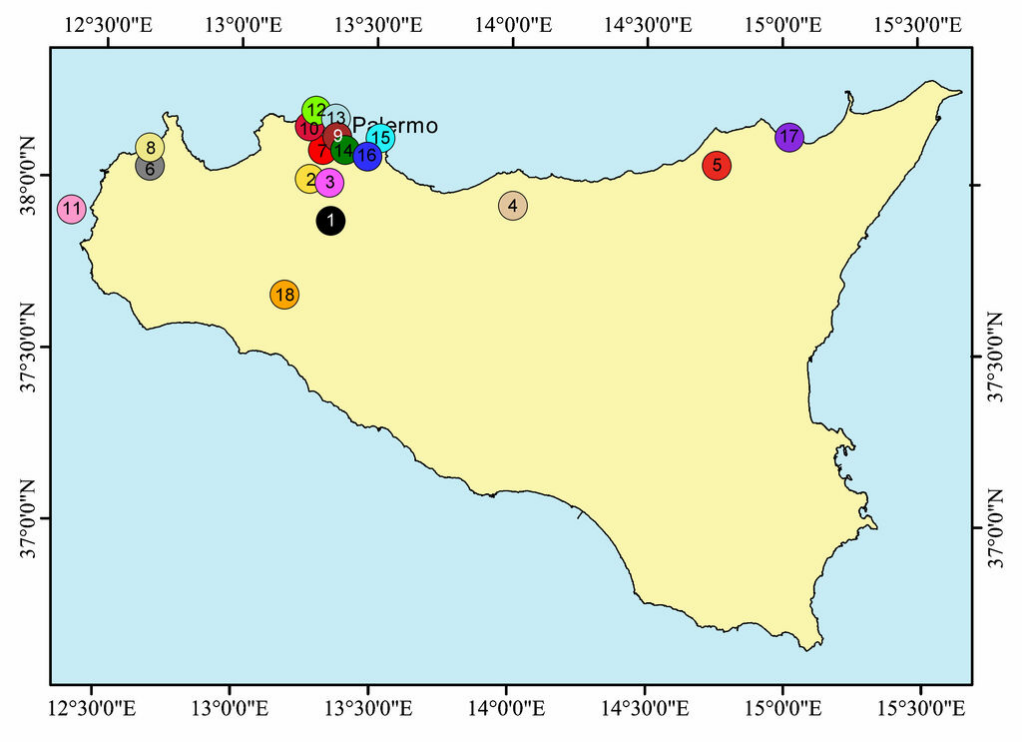
Distribution of the sampled populations. Population codes according to Table 1.

The population from the Rocche del Crasto was compared with four other populations of *C.busambarensis* and with 13 populations of other Sicilian species of the *C.busambarensis* complex. The studied material and its collection localities are reported in Table 1 and Fig. 1.

For the statistical analysis, specimens of the population of *Centaurea* from Rocche del Crasto were newly collected, voucher specimens being deposited in PAL and PAL-Gr; data published in Domina et al. (2017) for the other specimens of the *Centaureabusambarensis* complex were used. The methodology is the same as in Domina et al. (2017): 10 measurements were taken for each quantitative character of at least 10 different plants and five from Pizzolungo, given the small population (Suppl. material 1).

As reported in Guarino and Rampone (2006), the original descriptions of the taxa involved were used to draw up a list of characters of potential diagnostic suitability. A total of 26 characters were used: 19 continuous quantitative, four discrete quantitative and three qualitative.

**19 Continuous quantitative characters (mm)**

1.- Whole plant, height

2.- Rosette leaves, length

3.- Rosette leaves, width

4.- Lower stem leaves, length

5.- Lower stem leaves, width

6.- Upper stem leaves, length

7.- Upper stem leaves, width

8.- Apical lobe of rosette leaves, length

9.- Apical lobe of rosette leaves, width

10.- Lateral lobes of rosette leaves, length

11.- Lateral lobes of rosette leaves, width

12.- Capitula, length

13.- Capitula, width

14.- Median phyllaries, length

15.- Median phyllaries, width

16.- Appendage fimbriae of middle phyllaries, length

17.- Inner cypsela, length

18.- Inner cypsela, width

19.- Pappus of inner cypselas, length

**4 Discrete quantitative characters**

20.- Lobes of lower steam leaves, number

21.- Intermediate pinnulae of lower stem leaves, number

22.- Capitula per stem, number

23.- Appendage fimbriae of middle phyllaries, number

**3 Qualitative characters**

24.- Leaf shape (Pinnatisect / Bipinnatisect)

25.- Leaf indumentum (Glabrous / Subglabrous / Tomentose)

26.- Fimbriae colour (Pale / Dark)

Following Boyd (2002), Peruzzi et al. (2015) and Giovino et al. (2015), a principal component analysis (PCA) and a discriminant analysis (DA) were performed, both on all the 18 populations belonging to the whole *C.busambarensis* complex and on the five populations identified as *C.busambarensis*.

**Figure 2.**
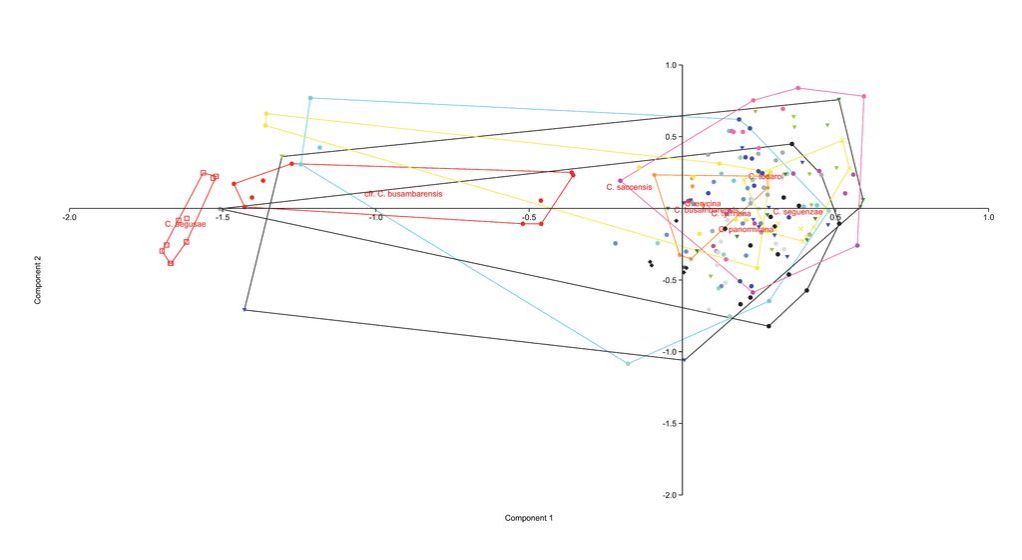
Principal component analysis based on the 19 continuous morphological characters, with groups corresponding to the 18 studied populations. PC1: Eigenvalue 0.340, % variance 37.00; PC2: Eigenvalue 0.130, % variance 14.11.

**Figure 3.**
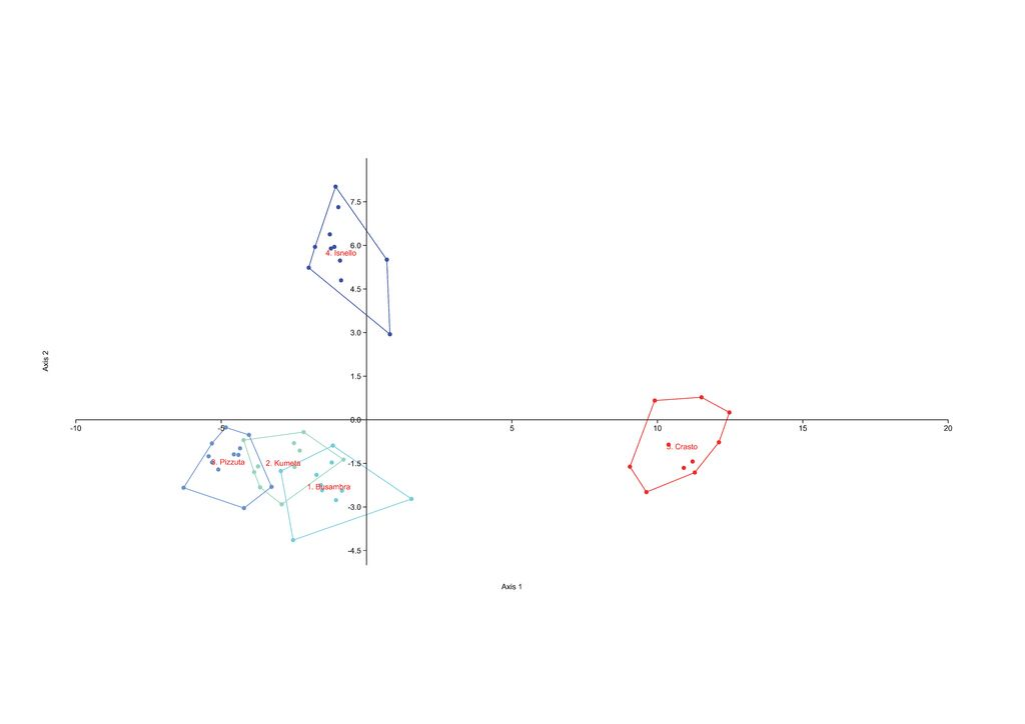
Principal component analysis, based on the 19 continuous morphological characters, with groups corresponding to the four populations of *Centaureabusambarensis* and of Rocche del Crasto. PC1: Eigenvalue 0.373, % variance 45.90; PC2: Eigenvalue 0.131, % variance 16.13.

**Figure 4.**
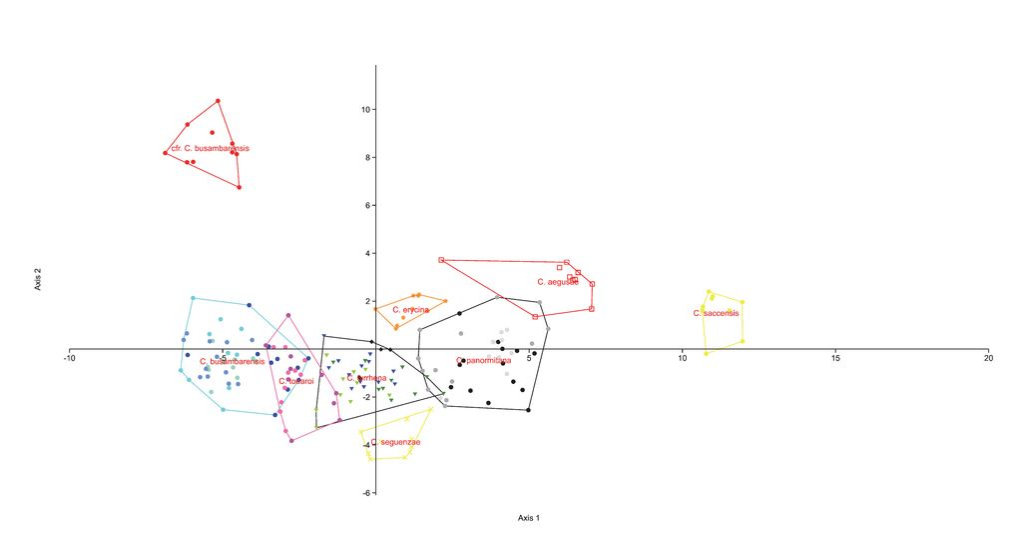
Discriminant analysis, based on the 26 considered morphological characters with groups corresponding to the nine studied taxa. Axis 1: Eigenvalue 19.199, % variance 52.69; Axis 2 Eigenvalue 6.094, % variance 16.72.

**Figure 5.**
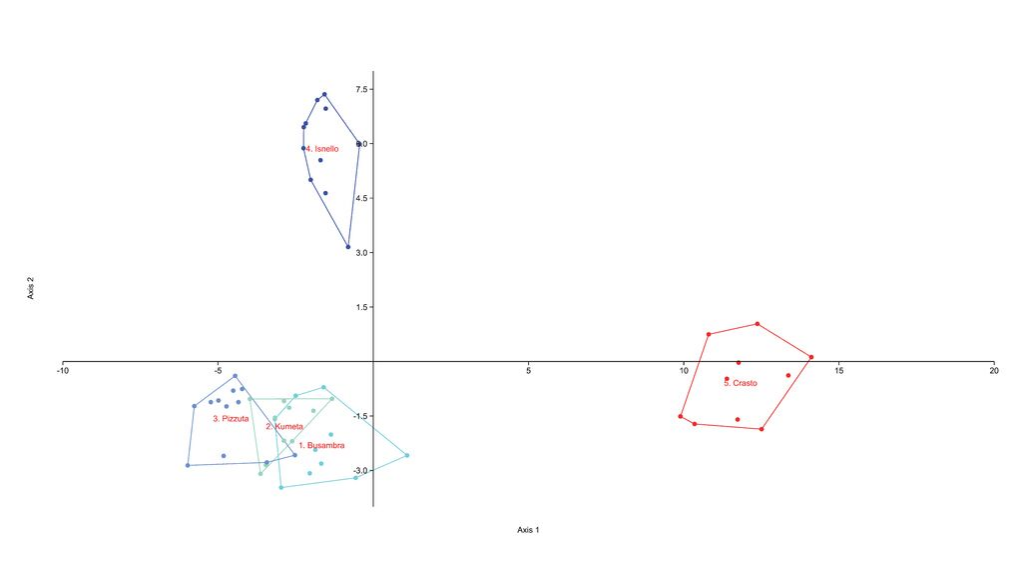
Discriminant analysis, based on the 26 considered morphological characters with groups corresponding to the four populations of *Centaureabusambarensis* and of Rocche del Crasto. Axis 1: Eigenvalue 32.434, % variance 71.51; Axis 2 Eigenvalue 9.830, % variance 21.67.

The PCAs (Figs 2, 3) were based on logarithmic values of the continuous quantitative characters. The DAs, with the individuals a priori assigned to the postulated groups, were performed on quantitative and qualitative characters (Figs 4, 5). Each continuous quantitative character was also subjected to univariate analysis (ANOVA or a Kruskal–Wallis test, with corrections for multiple comparisons, Pearson correlation coefficients, Tukey HSD test and Bonferroni, respectively), using PAST version 4.11 (Hammer et al. 2001 and Hammer 2022). The range of each continuous numerical character was represented using box-and-whiskers plots (Suppl. material 2).

## Data resources

Univariate analysis of all continuous morphological characters of the *Centaureabusambarensis* populations (Suppl. material 2) shows little variation. Isnello is the population that shows a higher number of outlier values in different characters. The quantitative characters that better characterise the population of Rocche del Crasto from the others are: the lower stem leaves width (35-40 vs. 17-35 mm); the upper stem leaves length (21-35 vs. 43-100 mm); the median phyllaries, width larger (4-4.2 vs. 3-4 mm) and the cypsela length (4.4-5.0 vs. 3.5-4.1 mm).

The PCA done on the complete dataset of 18 populations (Fig. 2) discriminates clearly only *C.aegusae*, the other taxa showing partial overlap. The DA on the complete dataset of 18 populations assigned to nine taxa (Fig. 4) discriminates better the groups, but a partial overlap between *C.busabarensis*, *C.todaroi*, *C.tyrrhena* and *C.panormitana* remains. A total of 98.4% of the individuals were correctly classified by DA to their *a priori* assigned taxon (Suppl. material 3) or 91.49% with the Jackknife method; see Osuji et al. (2013). The population from Rocche del Crasto is clearly differentiated. The PCA done only on *C.busambarensis* and the population from the Rocche del Crasto (Fig. 3) shows a complete overlap of the populations from Pizzuta, Kumeta and Busambra and only two individuals from Rocche del Crasto overlapping with the population from Busambra. The DA done only on *C.busambarensis* and the population from the Rocche del Crasto (Fig. 5) shows a partial overlap of the populations from Busambra, Kumeta and Pizzuta, a partial discrimination of the population of Isnello and a complete discrimination of the population from Rocche del Crasto. A total of 96.23% of the individuals were correctly classified by DA to their *a priori* assigned taxon (Suppl. material 3) or 67.92% with the Jackknife method; see Osuji et al. (2013). The initial hypothesis that the population from the Nebrodi Mountains is sufficiently distinct from the other taxa to deserve recognition at the species level is thus supported by statistical means. Therefore, we describe it as a new species named *Centaureavaldemonensis*.

## Taxon treatments

### Centaurea valdemonensis

Domina, Di Grist., Barone sp. nov.

5215FFC3-A1FB-586C-A0C3-AF414E7A3DDA

#### Materials

- **Type status:** Holotype. **Occurrence:** occurrenceID: 650C4F94-5407-55F7-BB94-EB377974DB39; recordedBy: Domina G., Di Gristina E.; **Taxon:** scientificName: *Centaureavaldemonensis* Domina, Di Grist., Barone; **Location:** country: Italy; stateProvince: Sicily; locality: Nebrodi Mountains, Rocche del Crasto; verbatimElevation: 1280 m a.s.l.; decimalLatitude: 38.013182; decimalLongitude: 14.737629; geodeticDatum: WGS84; **Event:** year: 2022; month: 6; day: 24; habitat: crevices of limestone rocks; **Record Level:** institutionCode: PAL109753; basisOfRecord: PreservedSpecimen
- **Type status:** Isotype. **Occurrence:** occurrenceID: B5EFFA30-8640-53BA-978E-BF71E39C29A2; recordedBy: Domina G., Di Gristina E.; **Taxon:** scientificName: *Centaureavaldemonensis* Domina, Di Grist., Barone; **Location:** country: Italy; stateProvince: Sicily; locality: Nebrodi Mountains, Rocche del Crasto, crevices of limestone rocks; verbatimElevation: 1280 m a.s.l.; decimalLatitude: 38.013182; decimalLongitude: 14.737629; geodeticDatum: WGS84; **Event:** year: 2022; month: 6; day: 24; habitat: crevices of limestone rocks; **Record Level:** institutionCode: PAL-Gr; basisOfRecord: PreservedSpecimen
- **Type status:** Isotype. **Occurrence:** occurrenceID: B0670CE1-DD18-5910-98BD-A734E50B28E2; recordedBy: Domina G., Di Gristina E.; **Taxon:** scientificName: *Centaureavaldemonensis* Domina, Di Grist., Barone; **Location:** country: Italy; stateProvince: Sicily; locality: Nebrodi Mountains, Rocche del Crasto, crevices of limestone rocks; verbatimElevation: 1280 m a.s.l.; decimalLatitude: 38.013182; decimalLongitude: 14.737629; geodeticDatum: WGS84; **Event:** year: 2022; month: 6; day: 24; habitat: crevices of limestone rocks; **Record Level:** institutionCode: SAF100085; basisOfRecord: PreservedSpecimen

#### Description

**Figure 6.**
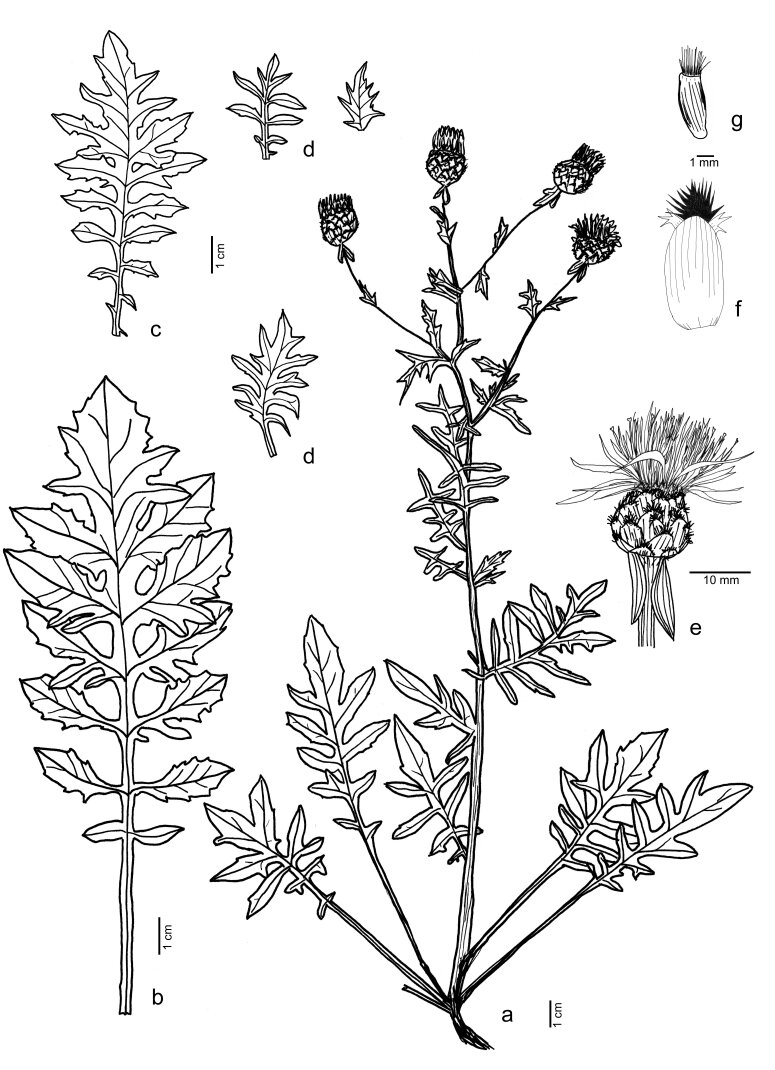
*Centaureavaldemonensis* Domina et al. **a** habit; **b** rosette leaf; **c** stem leaf; **d** branch leaves; **e** capitulum; **f** phyllary; **g** cypsela (drawn by G. Domina from the original material).

Perennial herb up to 50 cm, rosette-forming. Stem erect, white tomentose, with few branches above. Rosette leaves lyrate, 1–2 pinnatisect, white tomentose to arachnoid-hairy 9–30 cm long, 3–12 cm large. Cauline leaves 1–2 pinnatisect, with sinuate margins, white tomentose, 4–7 mm long, 3–4 mm large. Branch leaves entire, 5–10 mm × 3–6 mm. Capitula in clusters of 2–7. Peduncles 1–3 mm wide, with sparse leaves. Involucre ovoid, 11–15 × 11–16 mm; bracts ovate-lanceolate, glabrescent to arachnoid-hairy, with 7–9 nerves on the back. Appendages dark brown to black, shortly decurrent at the base, fimbriate. Appendages below the fimbria, with a 1–1.5 mm wide margin. Fimbriae 6–9 on each side, 1–2 mm long. Florets pink-violet, 12–18 mm long. Achenes light brown, 3.8–4.7 mm long, 1.5–2.1 mm wide. Pappus white, 1.5–2.0 mm long. (Fig. 6).

#### Diagnosis

Herba perennis, tomentosa, foliis 1-2 pinnatipartitis, incanis. Corymbus 2-7 capitulis; involucra ovata, 11–15 × 11–16 mm. Appendices fuscae vel nigrae; fimbriae 6–9 utroque latere, 1–2 mm longae. Flosculi roseo-lilacini; achenia luteo-brunnea 3.8–4.7 × 1.5–2.1 mm; pappus albus 1.5–2 mm longus.

#### Etymology

The specific epithet refers to the “Valdemone Mountains”, the name used since Middle Ages up to the 19^th^ Century for the NE Sicilian range where the species here described was found.

#### Distribution

As known so far, *Centaureavaldemonensis* occurs in a single population northeast Sicily, on the Nebrodi Mountains; but it is not excluded that the mountain complex may host other subpopulations.

#### Ecology

The known locality is found between 1,200 and 1,300 m a.s.l. Like other representatives of the *Centaureabusambarensis* complex, *C.valdemonensis* occurs on carbonate rocky habitat, with *Anthemiscupaniana* Nyman, *Athamantasicula* L., *Hyoserisradiata* L., *Saxifragagranulata* L., *Sedumhispanicum* L., *Seneciobalansae* Boiss. & Reut., TeucriumchamaedrysL.subsp.chamaedrys, TeucriumflavumL.subsp.flavum etc.

#### Conservation

The population of Rocche del Crasto includes about 300 mature individuals and extends for about 7000 m^2^. The plants that grow in the lower part of the cliff are subject to cow grazing.

#### Biology

Hemicryptophyte rosulate with chasmophyte habit, flowering and fruiting from June to August.

#### Taxon discussion

**Figure 7.**
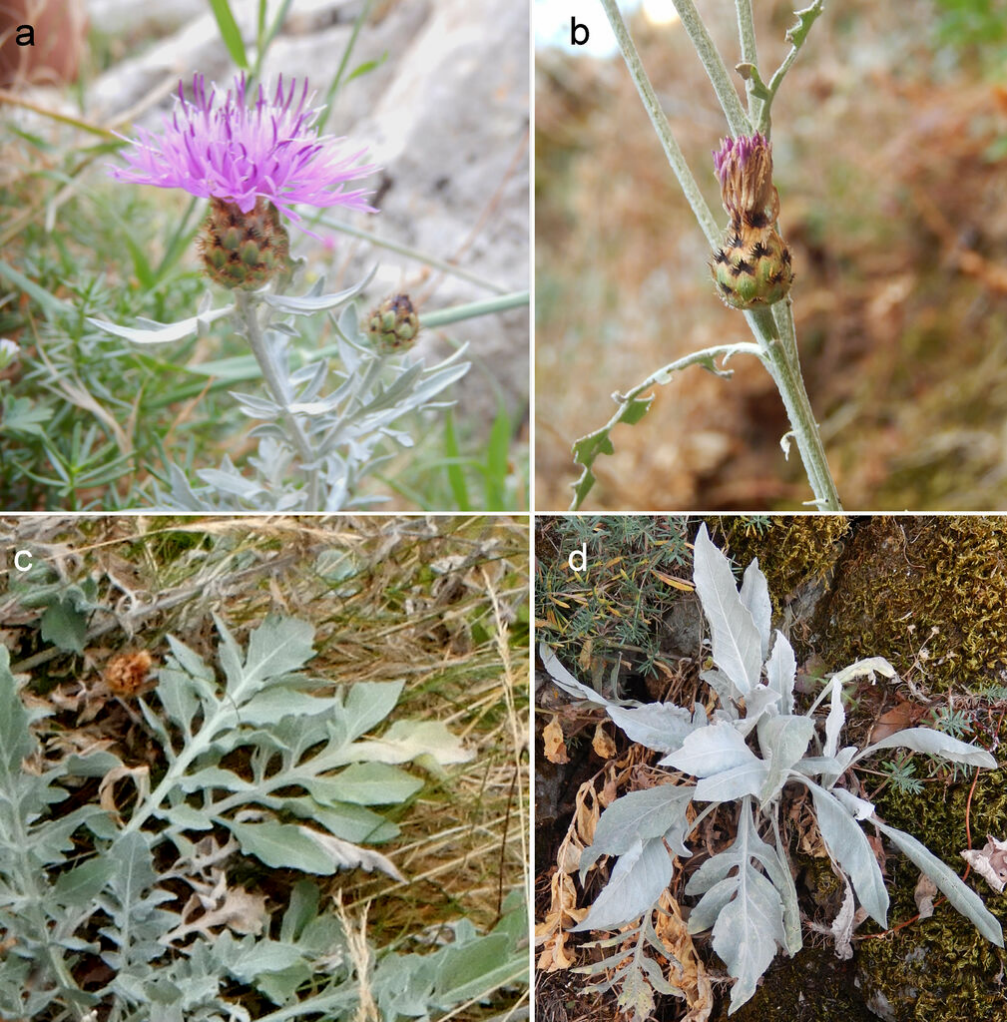
Capitulum in *Centaureavaldemonensis* (**a**) and *C.busambarensis* (**b**); rosette leaves in *C.valdemonensis* (**c**) and *C.busambarensis* (**d**).

The new species here described is well differentiated from the other species of the *Centaureabusambarensis* complex (Table 2). The most related species is *C.busambarensis*. The two species are, anyway, easily distinguishable by the shape of rosette leaves 1-2 pinnatisect with apical lobe slashed in *C.valdemonensis* and one pinnatisect with apical lobe almost entire in *C.busambarensis* (*Fig. 7*). In addition, the appendage of the median capitula bracts is shorter (2 mm) with shorter fimbriae (1–2 mm long) in *C.valdemonensis* than in *C.busambarensis* (3 mm long, with fimbriae 2.5–3 mm long) (Fig. 7). The shape of the rosette leaves is similar, in some ways, to that of *C.tauromenitana*, endemic to the east coast of Sicily (0–600 m a.s.l.), but not belonging to the *C.busambarensis* complex by having the yellow flowers in very large capitula with brown appendages and glabrescent habitus.

## Discussion

The Mediterranean area, despite being floristically well known, still reserves noteworthy taxonomic novelties that can be highlighted by the targeted study of the territory and with the support of statistical analysis. This discovery raises the number of species belonging to the *Centaureabusambarensis* group to nine, confirming the importance of Sicily in the evolution of this complex, as stated by Hilpold et al. (2011). Hilpold et al. (2014), studying nuclear and chloroplast DNA regions for most of the described species of the *Centaurea* group using phylogenetic and network approaches, concluded that the delimitation between at least some of the many described species is questionable. Integrated taxonomic studies that include small isolated populations, such as *C.valdemonensis*, can give important information to resolve this phylogenetic problem.

## Supplementary Material

XML Treatment for Centaurea valdemonensis

91F254F7-F812-542E-9699-25797CD17369 10.3897/BDJ.10.e91505.suppl1 Supplementary material 1 Morphological characters used for the statistical analysis (mean in mm) Data type morphological File: oo_750661.pdf https://binary.pensoft.net/file/750661 Domina G, Di Gristina E, Barone G

33D2E6E7-99C6-5532-9B04-0B850D32FA82 10.3897/BDJ.10.e91505.suppl2 Supplementary material 2 Plots of the 19 continuous numeric characters Data type graphs File: oo_749764.pdf https://binary.pensoft.net/file/749764 Domina G, Di Gristina E, Barone G

6714D511-1070-5951-8E6B-6A5D39AAE32D 10.3897/BDJ.10.e91505.suppl3 Supplementary material 3 Confusion matrices of the Discriminant Analyses Data type tables File: oo_721968.pdf https://binary.pensoft.net/file/721968 Domina G, Di Gristina E, Barone G

## Figures and Tables

**Table 1.** Source of sampled populations.

**Taxon and populations**	**Elevation (m a.s.l.)**	**Coordinates (WGS84, decimal degrees)**
***Centaureabusambarensis***		
1. Busambra	1,300	37.854759° N 13.415954° E
2. Kumeta	930	37.970513° N 13.256235° E
3. Pizzuta	1,030	37.997086° N 13.246412° E
4. Isnello, castel	570	37.945732° N 14.005906° E
5. Rocche del Crasto	1,280	38.013182° N 14.737629° E
***C.erycina***		
6. Erice	720	38.035189° N 12.591671° E
***C.panormitana***		
7. Punta Mastrangelo	850	38.064298° N 13.243363° E
8. Pizzolungo	40	38.063512° N 12.570256° E
9. Mt. Pellegrino, S slope	150	38.153465° N 13.360442° E
10. Mt. Pecoraro	750	38.162269° N 13.123313° E
***C.aegusae***		
11. Favignana, Mt. Santa Caterina	100	37.920730° N 12.307299° E
***C.thyrrena***		
12. Mt.Gallo	500	38.218151° N 13.311306° E
13. Mt. Pellegrino, N slope	200	38.187822° N 13.335711° E
14. Mt. Grifone	250	38.071647° N 13.370249° E
***C.todaroi***		
15. Mongerbino	30	38.110688° N 13.538341° E
16. Mt. Catalfano	100	38.114900° N 13.513798° E
***C.seguenzae***		
17. Cape Tindari	20	38.137394° N 15.052898° E
***C.saccensis***		
18. Tardara Gorges	130	37.614346° N 13.052547° E

**Table 2.** Diagnostic characters between the species of the *Centaureabusambarensis* complex. VAL: *C.valdemonensis*; BUS: *C.busambarensis*; ERY: *C.erycina*; PAN: *C.panormitana*; AEG: *C.aegusae*; THY: *C.thyrrena*; TOD: *C.todaroi*; SEG: *C.seguenzae*; SAC: *C.saccensis*.

	**VAL**	**BUS**	**ERY**	**PAN**	**AEG**	**THY**	**TOD**	**SEG**	**SAC**
**Rosette leaves shape**	1–2 pinnatisect	entire or 1 pinnatisect	1–2 pinnatisect	1–2 pinnatisect	2-pinnatisect	2-pinnatisect	2-pinnatisect	1–2 pinnatisect	1–2 pinnatisect
**Apical lobe of rosette leaves shape**	pinnatisect	almost entire	pinnatisect	almost entire	pinnatisect	almost entire	pinnatisect	almost entire	almost entire
**Apical lobe of rosette leaves,width (mm)**	10–30	8–20	5–18	5–18	3–5	5–15	4–20	5–18	10–14
**Leaf indumentum**	white tomentose	white tomentose	white tomentose	white tomentose	white tomentose	glabrescent, rarely somewhat arachnoid	glabrescent, rarely somewhat arachnoid	glabrescent, rarely somewhat arachnoid	white tomentose
**No. of capitula per stem**	2–7	2–9	2–21	1–7	8–20	2–14	2–20	1–6	2–7
**Involucre shape**	ovoid	ovoid-globose	ovoid-globose	ovoid-globose	ovoid	ovoid	ovoid	ovoid	ovoid
**Appendages**	dark brown to black	dark brown to black	dark to light brown	dark to light brown	dark brown	dark to light brown	light brown	light brown	dark brown to black
**No. of fimbriae on each side**	6–9	6–9	6–8	5–7	5–7	5–9	5–9	4–7	6–9
**Fimbriae of phyllaries, length (mm)**	1–2	2.5–3	2–3	1–2	1.5–2	1–2	1–2	1–2	2–3
**Pappus/Cypsela**	<1	<1	<1	≈ 1	≈ 1	≈ 1	<1	≈ 1	>1
