# Supplementary material for: A new species within the Centaureabusambarensis complex (Asteraceae, Cardueae) from Sicily
Source: Biodivers Data J. 2022 Oct 6;10:e91505. doi: 10.3897/BDJ.10.e91505 (PMC9836586; doi:10.3897/BDJ.10.e91505)
Supplement: Supplementary material 1 — Morphological characters used for the statistical analysis (mean in mm) [file bdj-10-e91505-s001.pdf]

Suppl. Material 1. Morphological characters used for the statistical analysis (mean in mm).

Population	Taxon	1.- Whole plant, height	2.- Rosette leaves, length	3.- Rosette leaves, width	4.- Lower stem leaves, length	5.- Lower stem leaves, width	6.- Upper stem leaves, length	7.- Upper stem leaves, width	8.- Apical lobe of rosette leaves, length	9.- Apical lobe of rosette leaves, width	10.- Lateral lobes of rosette leaves, length	11.- Lateral lobes of rosette leaves, width	12.- Capitula, length	13.- Capitula, width	14.- Median phyllaries, length	15.- Median phyllaries, width	16.- Appendage fimbriae of middle phyllaries, length	17.- Inner cypsela, length	18.- Inner cypsela, width	19.- Pappus of inner cypselas, length	20.- Lobes of lower steam leaves, number	21.- Intermediate pinnulae of lower stem leaves, number	22.- Capitula per stem, number	23.- Appendage fimbriae of middle phyllaries, number	24.- Leaf shape (0. Pinnatisect / 1. Bipinnatisect)	25.- Leaf indumentum (1. Glabrous / 2. Subglabrous / 3. Tomentose)	26.- Fimbriae colour (0. Dark / 1. Pale)
1. Busambra	*C. busambarensis*	260	110	40	40	10	90	15	20	8	30	3	9	9	6	3	2	3,9	2,1	1,3	7	6	2	13	1	3	0
1. Busambra	*C. busambarensis*	460	110	40	85	40	110	30	45	25	25	14	14	16	5	4	2	4	2,1	1,3	5	3	9	12	1	3	0
1. Busambra	*C. busambarensis*	320	195	60	45	25	100	40	45	25	30	8	16	16	5	3	2	4	2,1	1,4	4	3	3	12	1	3	0
1. Busambra	*C. busambarensis*	755	190	60	70	30	100	40	43	21	30	11,5	16	18	8	4	2	4	2	1,3	5	0	6	17	1	3	0
1. Busambra	*C. busambarensis*	480	225	70	55	28	70	15	35	20	45	5	13	10	5	5	2	4	1,7	1,7	5	3	5	25	1	3	0
1. Busambra	*C. busambarensis*	340	210	45	65	22	115	25	30	20	35	25	16	13	3	2,5	2	4	1,7	1,8	4	4	1	17	1	3	0
1. Busambra	*C. busambarensis*	500	150	40	70	40	55	25	25	10	30	7	16,5	12	7	4	2	4	1,7	1,8	5	4	4	17	1	3	0
1. Busambra	*C. busambarensis*	500	170	35	70	35	55	18	35	20	30	7	15,5	13	5	4	2	4	1,8	1,8	4	6	3	17	1	3	0
1. Busambra	*C. busambarensis*	430	240	60	65	30	80	20	33	20	35	16,5	12,5	11	4	4	2	4	1,8	1,8	7	0	2	14	1	3	0
1. Busambra	*C. busambarensis*	395	170	42	65	30	115	35	33	14	25	8	15	13	4	4	2	4	1,8	1,8	5	0	3	19	1	3	0
2. Kumeta	*C. busambarensis*	560	88	21	102	56	57	10	45	22	30	7,5	17	14	5	3	1,8	4	1,8	1,8	5	5	9	20	1	3	0
2. Kumeta	*C. busambarensis*	675	185	60	100	25	75	30	50	25	35	7	15	11	5	4	1,5	4	1,7	1,8	4	3	4	15	1	3	0
2. Kumeta	*C. busambarensis*	460	135	30	50	40	55	20	25	10	30	7	16,5	12	7	4	2	4	1,8	1,8	5	4	4	17	1	3	0
2. Kumeta	*C. busambarensis*	260	180	38	25	15	60	10	20	5	25	7	16	11	5	4	2,5	3,6	1,5	1,5	6	3	8	16	0	3	0
2. Kumeta	*C. busambarensis*	240	60	20	20	10	40	10	10	3	11	6,5	13	11	4	3	2	3,6	1,5	1,5	5	3	2	15	0	3	0
2. Kumeta	*C. busambarensis*	255	80	27	53	20	55	14	18	10	11	7	12	12	5	4	2,5	3,6	1,5	1,5	4	4	1	13	0	3	0
2. Kumeta	*C. busambarensis*	250	120	20	45	20	85	17	25	5	20	11	13	11	6	4	1,5	3,6	1,5	1,5	5	4	4	12	0	3	0
2. Kumeta	*C. busambarensis*	240	125	30	30	18	58	10	20	18	13	7	12	13	4	3	2	3,6	1,5	1,5	5	4	2	12	1	3	0
2. Kumeta	*C. busambarensis*	515	195	55	58	23	105	23	35	20	33	5,5	14	10	5	4	2	3,6	1,5	1,5	6	4	5	14	0	3	0
2. Kumeta	*C. busambarensis*	520	185	25	130	35	55	10	35	20	30	7	15	13	5	4	2	4	1,8	1,8	4	6	3	17	1	3	0
3. Pizzuta	*C. busambarensis*	330	155	60	68	24	65	22	35	16	25	8,5	12	11	6	4	3	3,6	1,5	1,5	5	4	5	16	0	3	0
3. Pizzuta	*C. busambarensis*	340	150	25	50	20	100	20	30	8	20	8	12	10	6	4	2,5	3,6	1,5	1,5	6	4	2	15	0	3	0
3. Pizzuta	*C. busambarensis*	300	150	45	40	22	95	30	40	20	20	13	13	10	4	3	2	3,7	1,5	1,5	4	3	2	16	1	3	0
3. Pizzuta	*C. busambarensis*	260	145	45	28	20	90	28	40	20	25	7,5	12	8	4	3	2	3,6	1,5	1,5	4	4	3	16	0	3	0
3. Pizzuta	*C. busambarensis*	390	140	42	40	22	65	15	40	18	25	10	16	12	6	4	2	3,6	1,5	1,5	4	4	5	19	0	3	0
3. Pizzuta	*C. busambarensis*	380	95	25	50	20	50	15	17	5	13	11	13	12	6	3	2,5	3,6	1,5	1,5	5	4	2	15	1	3	0
3. Pizzuta	*C. busambarensis*	340	110	35	27	14	35	15	24	15	16	13,5	14	13	5	4	2	3,6	1,5	1,5	5	3	5	15	0	3	0
3. Pizzuta	*C. busambarensis*	290	170	40	45	20	110	28	35	10	24	13,5	14	12,5	5	4	2	3,7	1,5	1,5	5	4	2	15	0	3	0
3. Pizzuta	*C. busambarensis*	180	180	35	36	10	43	14	25	10	22	18	13	12	4	3	2	3,6	1,5	1,5	6	3	3	15	0	3	0
3. Pizzuta	*C. busambarensis*	540	140	30	45	20	65	20	35	10	20	11	14	13	5	4	2	3,6	1,5	1,5	7	4	12	14	1	3	0
3. Pizzuta	*C. busambarensis*	220	180	40	35	18	65	10	35	13	25	14,5	13	10	4	3	3	3,6	1,5	1,5	5	3	7	16	0	2	0
3. Pizzuta	*C. busambarensis*	230	145	20	33	20	65	12	25	6	13	12	13	12	5	3	2	3,6	1,5	1,5	6	2	7	14	1	3	0
4. Isnello	*C. busambarensis*	955	175	65	57	30	85	30	45	12	35	4,5	15	15	6	4,5	3	4,1	1,6	2,2	8	4	7	15	1	3	0
4. Isnello	*C. busambarensis*	785	200	60	100	35	80	30	30	10	35	4,5	14	15	6	5	2	4	1,5	2,2	5	4	5	15	1	3	0
4. Isnello	*C. busambarensis*	440	210	50	33	10	85	30	27	7	35	8	10	7	4	3	2	4,1	1,6	2,3	5	4	11	17	1	3	0
4. Isnello	*C. busambarensis*	550	150	20	50	12	75	10	30	10	35	3,5	12	12	8	3	1	4	1,4	2,3	5	4	2	15	1	3	0
4. Isnello	*C. busambarensis*	345	170	50	40	35	80	30	40	17	25	9,5	14	12	6	3	2	4	1,5	2,2	6	4	4	19	1	3	0
4. Isnello	*C. busambarensis*	770	180	85	40	35	85	15	35	10	40	18	13	13	5	4	2,5	4	1,6	2,3	7	4	10	18	1	3	0
4. Isnello	*C. busambarensis*	440	190	80	40	35	85	15	35	10	40	19	15	10	6	4	2	4	1,5	2,3	6	4	18	16	1	3	0
4. Isnello	*C. busambarensis*	380	190	82	50	30	80	20	30	10	35	14	16	22	8	4	2,5	4,1	1,3	2,3	5	4	4	17	1	2	0
4. Isnello	*C. busambarensis*	360	200	85	50	30	80	20	30	10	35	14	18	22	6	3	2	4	1,6	2,3	5	4	7	11	1	3	0
4. Isnello	*C. busambarensis*	390	130	30	60	25	85	25	30	10	15	4	15	15	5	3	2	4	1,6	2,3	5	4	1	11	1	3	0
4. Isnello	*C. busambarensis*	190	140	35	95	35	80	25	30	10	20	13	13	15	5	3	2	4,1	1,3	1,6	5	5	3	12	1	3	0
5. Crasto	cfr. *C. busambarensis*	270	95	30	45	40	35	18	15	10	26	10	15	11	4	4	1,6	4,7	2,1	2	11	1	4	13	1	3	0
5. Crasto	cfr. *C. busambarensis*	300	95	42	60	40	35	20	15	7	14	6	15	15	6	4	2,2	5	1,8	1,6	13	1	6	13	1	3	0
5. Crasto	cfr. *C. busambarensis*	410	130	44	60	44	32	18	30	13	18	7	13	13	5	5	2,3	4,5	2	1,7	11	0	4	11	0	3	0
5. Crasto	cfr. *C. busambarensis*	430	120	42	70	36	24	16	16	10	16	6	12	14	4	4	2,1	4,6	1,7	1,7	11	0	4	13	1	3	0
5. Crasto	cfr. *C. busambarensis*	420	160	42	120	40	34	18	35	18	22	9	11	15	6	5	2,5	4,7	1,6	1,8	11	0	3	9	1	3	0
5. Crasto	cfr. *C. busambarensis*	460	120	42	95	40	21	16	35	30	22	12	13	13	6	4	2,5	4,6	1,6	1,6	11	1	4	9	1	3	0
5. Crasto	cfr. *C. busambarensis*	410	150	44	110	36	28	24	38	19	27	8	12	15	4	4	2,1	4,6	1,7	1,9	11	1	4	11	1	3	1
5. Crasto	cfr. *C. busambarensis*	410	130	38	100	32	22	20	25	12	14	6	13	15	5	4	2,5	4,7	1,7	1,7	13	0	4	9	0	3	0
5. Crasto	cfr. *C. busambarensis*	380	120	36	80	35	33	16	16	11	14	6	13	14	4	4	2	4,5	1,6	1,6	11	0	9	11	0	3	1
5. Crasto	cfr. *C. busambarensis*	350	95	40	60	40	35	28	20	11	18	9	14	14	4	4	2,5	4,4	1,8	1,6	11	1	5	11	1	3	0
6. Erice	*C. erycina*	350	150	42	56	18	80	45	26	17	21	5,5	16	14	5	3	2	4,2	2	2,5	5	3	6	13	0	3	1
6. Erice	*C. erycina*	200	155	40	35	15	85	52	25	5	20	6,5	11	13	5	3	2	4,5	1,9	2,5	6	3	3	12	0	3	0
6. Erice	*C. erycina*	245	150	37	40	15	120	25	25	10	22	12,5	17	18	6	4	3	4,4	1,9	2,5	5	3	7	11	0	3	0
6. Erice	*C. erycina*	300	140	70	40	20	60	20	20	10	25	4	14	21	5	3	2	4	1,9	2,4	7	3	2	11	0	3	0
6. Erice	*C. erycina*	275	145	75	50	30	85	50	25	10	25	10,5	14	25	4	3	3	4,3	2	2,4	6	2	5	14	0	2	0
6. Erice	*C. erycina*	495	145	68	60	35	80	47	25	10	25	7,5	15	15	5	3	2	4,5	2	2,5	6	5	6	11	0	3	0
6. Erice	*C. erycina*	250	158	41	38	18	118	27	28	14	19	13	17	18	6	4	3	4,5	1,9	2,5	5	3	8	12	0	3	0
6. Erice	*C. erycina*	320	152	45	54	27	96	34	26	12	21	12	16	15	5	3	2	4,5	2	2,4	6	5	6	11	0	3	0
6. Erice	*C. erycina*	280	138	65	42	24	93	29	24	14	23	9,5	17	18	6	4	3	4,4	1,9	2,5	5	3	8	12	0	3	0
6. Erice	*C. erycina*	340	148	42	58	19	78	47	28	18	23	6	15	14	6	4	2,8	4,4	1,9	2,5	5	3	4	13	0	3	0
7. Mastrangelo	*C. panormitana*	440	118	35	35	10	39	10	21	4	25	3	14	15	5	2	2	4,4	1,8	3,6	5	6	4	13	1	3	0
7. Mastrangelo	*C. panormitana*	320	92	32	45	20	40	19	10	5	15	8	14	10	5	3	2,2	4,4	1,8	3,7	4	6	1	12	1	3	0
7. Mastrangelo	*C. panormitana*	540	124	34	52	20	45	9	23	5	18	4	16	12	9	2	2	4,5	1,8	3,7	6	5	2	10	0	3	0
7. Mastrangelo	*C. panormitana*	445	176	53	62	15	70	17	32	7	18	7	14	14	8	3	2	4,5	1,8	3,5	8	6	5	11	1	3	1
7. Mastrangelo	*C. panormitana*	790	180	60	112	35	75	12	28	7	20	8	17	14	7	3	1,5	4,4	1,8	3,7	7	7	2	14	1	2	0
7. Mastrangelo	*C. panormitana*	415	170	55	90	30	75	18	30	7	20	3	18	13	9	3	2	4,5	1,8	3,5	7	5	2	12	1	2	0
7. Mastrangelo	*C. panormitana*	380	110	30	50	35	85	22	15	5	16	11,5	16	16	9	4	2,1	4,5	1,9	3,5	4	4	5	12	1	2	0
7. Mastrangelo	*C. panormitana*	355	175	45	50	30	145	38	35	10	25	3,5	10	9	5	3	2	4,5	1,8	3,5	6	5	4	13	1	3	0
7. Mastrangelo	*C. panormitana*	360	250	65	50	30	74	20	30	10	40	1,5	16	15	7	3	1	4,4	1,8	3,5	6	5	1	14	1	2	1
7. Mastrangelo	*C. panormitana*	385	160	50	46	16	95	15	22	14	26	3	16	13	6	3	2	4,4	1,9	3,7	8	5	4	12	1	2	1
7. Mastrangelo	*C. panormitana*	600	235	65	65	20	119	22	26	14	36	7,5	17	11	8	3	2,4	4,4	1,8	3,6	8	5	2	10	0	2	1
7. Mastrangelo	*C. panormitana*	560	195	69	59	20	68	22	38	18	30	7	15	12	8	3	1,5	4,4	1,9	3,7	8	5	3	12	1	2	1
7. Mastrangelo	*C. panormitana*	340	120	40	85	20	85	19	24	13	18	3,5	18	14	7	3	1,8	4,5	1,8	3,6	5	6	1	12	1	3	1
7. Mastrangelo	*C. panormitana*	580	240	75	70	20	124	30	42	18	40	9	10	10	7	3	1	4,5	1,8	3,6	6	5	7	15	0	2	0
8. Pizzolungo	*C. tyrrhena*	230	130	65	25	18	90	20	18	7	20	7	14	12	5	2	1	4,6	1,4	2,6	6	3	2	12	1	2	0
8. Pizzolungo	*C. tyrrhena*	230	125	60	36	20	90	20	18	7	20	11	12	12	5	3	2	4,5	1,6	2,6	6	3	6	12	1	2	1
8. Pizzolungo	*C. tyrrhena*	270	130	32	20	18	100	20	15	5	20	5	13	11	5	4	2	4,6	1,4	2,6	6	3	6	13	0	2	0
8. Pizzolungo	*C. tyrrhena*	260	128	28	22	19	89	18	17	5	21	4,5	13	11	5	4	2	4,5	1,4	2,6	6	3	6	13	0	2	0
8. Pizzolungo	*C. tyrrhena*	255	119	48	25	18	98	27	18	7	20	8,5	14	12	5	2	1	4,6	1,5	2,5	6	3	2	12	1	2	0
9. Mt Pellegrino, S slope	*C. panormitana*	425	140	30	75	55	90	45	15	5	30	6	16	22	8	3,5	2	4,4	1,7	3	6	6	2	14	0	3	0
9. Mt Pellegrino, S slope	*C. panormitana*	370	100	30	70	50	75	25	10	5	10	11,5	12	12	6	4	2	4,4	1,8	3,6	6	6	6	11	0	3	0
9. Mt Pellegrino, S slope	*C. panormitana*	475	175	65	70	50	95	40	25	5	40	8	12	14	6	3	1	4,3	1,8	3,6	6	5	8	14	0	3	1
9. Mt Pellegrino, S slope	*C. panormitana*	360	160	60	80	65	85	25	15	5	30	16	14	16	6	4	1	4,6	1,8	3,7	6	3	9	13	0	3	1
9. Mt Pellegrino, S slope	*C. panormitana*	300	150	35	80	65	85	25	18	5	30	4	15	16	6	4	2	4,3	1,8	3,6	6	3	3	13	0	3	1
9. Mt Pellegrino, S slope	*C. panormitana*	240	150	40	80	65	80	20	15	5	28	4	16	16	7	4	2	3,8	1,8	3	6	3	2	14	0	3	0
9. Mt Pellegrino, S slope	*C. panormitana*	310	75	170	39	14	36	10	24	5	15	6	14	12	8	4	1,5	4,2	1,5	2,7	3	0	8	11	1	2	0
9. Mt Pellegrino, S slope	*C. panormitana*	600	160	60	90	65	80	25	22	5	20	9,5	12	16	8	3	2	4,4	1,5	2,8	4	7	7	15	0	2	0
9. Mt Pellegrino, S slope	*C. panormitana*	560	150	55	45	45	80	25	22	5	20	12	15	16	7	4	2	4,4	1,5	2,7	4	5	5	14	0	2	0
9. Mt Pellegrino, S slope	*C. panormitana*	420	160	58	45	50	80	25	25	5	20	19	13	16	7	4	1,8	4,4	1,6	2,8	4	5	13	13	0	2	0
9. Mt Pellegrino, S slope	*C. panormitana*	570	155	55	42	45	75	25	25	5	20	13	16	15	7	4	1	4,4	1,5	2,8	4	5	6	13	0	2	0
9. Mt Pellegrino, S slope	*C. panormitana*	560	162	52	45	45	95	27	20	5	25	18	16	14	8	4	2,2	4	1,8	2,6	7	6	12	15	0	2	0
9. Mt Pellegrino, S slope	*C. panormitana*	540	150	48	47	30	75	25	25	5	20	7,5	17	18	6	5	2	4,2	1,8	2,6	4	5	7	12	0	3	0
10. Mt Pecoraro	*C. panormitana*	305	115	23	30	25	65	12	35	15	40	2,5	15	13	3	3	1,8	4,8	1,9	3,6	4	7	7	11	1	3	0
10. Mt Pecoraro	*C. panormitana*	317	192	60	30	10	98	33	13	5	30	4,5	15	13	4	5	2	4,5	1,8	3,7	6	8	7	11	1	3	0
10. Mt Pecoraro	*C. panormitana*	320	155	47	25	28	105	35	26	8	20	15	13	13	5	4	1,5	4,4	1,8	3,6	6	5	4	14	1	3	0
10. Mt Pecoraro	*C. panormitana*	380	104	25	30	25	58	12	23	16	10	3	17	12	5	4	2	4,8	1,9	3,6	5	5	4	15	1	3	0
10. Mt Pecoraro	*C. panormitana*	290	185	41	27	25	78	21	20	19	20	15	14	12	6	4	2	4,4	1,8	3,7	5	5	6	8	1	3	0
10. Mt Pecoraro	*C. panormitana*	370	95	25	28	26	37	7	13	8	11	4	15	13	4	2	1	4,8	1,9	3,6	6	5	4	12	1	3	1
10. Mt Pecoraro	*C. panormitana*	405	120	30	28	28	50	10	23	9	12	5	18	15	5	3	1,5	4,5	1,7	3,6	5	5	8	12	1	3	1
10. Mt Pecoraro	*C. panormitana*	450	140	35	45	20	55	14	23	10	15	7,5	15	13	4	3	1,5	4,5	1,8	3,7	6	5	10	12	1	3	1
10. Mt Pecoraro	*C. panormitana*	400	100	28	30	22	54	12	20	14	10	4,5	17	13	5	4	2	4,3	1,8	3,6	5	5	6	15	1	3	0
10. Mt Pecoraro	*C. panormitana*	390	110	32	32	28	57	17	18	12	12	5	18	15	5	3	1,5	4,5	1,7	3,5	5	5	8	14	1	3	1
11. Favignana	*C. aegusae*	460	70	30	22	12	45	22	12	4	15	6	13	13	5	5	2	3,3	1,3	3	8	0	8	12	0	3	1
11. Favignana	*C. aegusae*	650	200	50	45	22	70	30	12	3	17	5	13	13	5	3	1	3,3	1,3	3,4	6	0	15	12	0	3	1
11. Favignana	*C. aegusae*	350	70	30	15	10	50	22	9	5	12	5,5	13	12	5	4	1	3,4	1,1	4	6	0	8	10	0	3	1
11. Favignana	*C. aegusae*	500	150	80	15	10	60	22	9	5	12	5,5	13	12	5	4	1	3,4	1,1	4	8	0	10	11	0	3	1
11. Favignana	*C. aegusae*	350	80	30	15	12	50	22	10	5	10	4,5	13	13	5	4	1	3,5	1,1	3,8	8	0	12	10	0	3	1
11. Favignana	*C. aegusae*	500	130	50	15	12	60	22	15	5	12	6	14	13	5	4	1	3,5	1,1	4	8	0	10	11	0	3	1
11. Favignana	*C. aegusae*	350	70	30	15	10	50	22	9	5	15	4,5	13	12	5	4	1	3,4	1,1	4	8	0	8	10	0	3	1
11. Favignana	*C. aegusae*	350	85	30	15	10	50	22	14	5	10	5,5	14	12	5	4	1	3,4	1,1	4	8	0	12	10	0	3	1
11. Favignana	*C. aegusae*	650	145	60	35	15	80	45	20	4	15	4,5	15	13	4	3	3	3,5	1,1	3,8	6	0	13	12	0	3	1
11. Favignana	*C. aegusae*	335	172	80	45	45	25	21	22	3	12	4,5	12	14	4	3	2	3,4	1,1	4	6	0	20	11	0	3	1
12. Mt Gallo	*C. tyrrhena*	520	180	50	42	20	60	21	35	15	30	13	13	13	8	3,5	1	3,8	1,6	2,4	7	3	4	12	1	2	1
12. Mt Gallo	*C. tyrrhena*	530	178	50	60	30	110	33	39	10	30	18	13	12	7	4,5	1	3,8	1,6	2,4	7	6	14	12	1	2	1
12. Mt Gallo	*C. tyrrhena*	705	160	54	85	55	65	17	35	15	30	23	16	15	9	4,5	2	3,9	1,6	2,6	7	5	10	14	1	1	1
12. Mt Gallo	*C. tyrrhena*	675	115	25	135	85	70	35	15	5	10	11	13	14	5	4	1,5	3,9	1,6	2,4	5	7	5	13	1	2	0
12. Mt Gallo	*C. tyrrhena*	620	150	60	60	35	110	35	35	15	30	21	13	13	5	3	2	3,9	1,6	2,4	5	4	13	16	1	2	0
12. Mt Gallo	*C. tyrrhena*	690	200	60	70	50	90	36	28	26	15	20	17	15	8	2,5	1,8	3,9	1,5	2,4	7	6	2	12	1	2	0
12. Mt Gallo	*C. tyrrhena*	510	220	75	80	25	110	28	20	10	40	9	14	14	4	2,5	2	3,8	1,5	2,4	6	6	5	14	1	2	0
12. Mt Gallo	*C. tyrrhena*	580	200	80	110	70	50	20	20	10	35	15	13	13	8	4	1,5	3,8	1,6	2,5	6	7	11	13	1	2	0
12. Mt Gallo	*C. tyrrhena*	320	85	30	35	15	45	20	10	5	17	9	13	13	4	3	2	3,6	1,4	2,4	5	5	5	12	0	2	0
12. Mt Gallo	*C. tyrrhena*	465	130	25	38	20	110	20	15	5	20	10,5	16	16	5	2	2	3,6	1,3	2	7	6	4	12	0	2	0
13. Mt Pellegrino, N slope	*C. tyrrhena*	610	285	85	90	70	170	70	45	15	50	15	14	14	6	3,5	1,5	3,8	1,7	3	6	5	4	15	0	2	0
13. Mt Pellegrino, N slope	*C. tyrrhena*	200	105	30	120	60	55	18	15	5	20	7,5	12	13	6	2	2	3,8	1,7	3	5	7	5	13	1	2	1
13. Mt Pellegrino, N slope	*C. tyrrhena*	565	100	28	235	60	60	14	15	5	25	22	10	10	6	4,5	1,5	3,8	1,7	3	5	8	3	13	1	3	1
13. Mt Pellegrino, N slope	*C. tyrrhena*	990	170	65	50	50	60	17	30	5	24	8	16	15	6	4,5	1,5	4,3	2	2,7	5	6	8	13	1	2	0
13. Mt Pellegrino, N slope	*C. tyrrhena*	500	175	20	25	20	60	14	10	4	25	10	11	16	5	3	2	4	2	2	7	4	6	14	0	2	0
13. Mt Pellegrino, N slope	*C. tyrrhena*	380	115	30	50	25	60	14	15	5	25	7,5	15	17	7	4	2	4,4	1,5	2,8	5	5	7	11	0	2	0
13. Mt Pellegrino, N slope	*C. tyrrhena*	325	180	75	57	20	65	20	16	5	20	11	14	14	6	4	2	3,8	1,6	2,4	6	6	6	10	0	2	0
13. Mt Pellegrino, N slope	*C. tyrrhena*	475	180	73	25	20	65	20	15	5	20	14	15	13	6	4	2	3,8	1,1	2,4	6	3	6	11	0	2	0
13. Mt Pellegrino, N slope	*C. tyrrhena*	300	110	45	122	63	57	19	18	5	21	8	12	13	6	2	2	3,9	1,7	3	5	7	5	13	1	3	1
13. Mt Pellegrino, N slope	*C. tyrrhena*	552	100	36	221	60	58	14	17	5	28	22	12	11	6	4,5	1,5	3,8	1,7	3	5	8	3	13	1	3	1
14. Mt Grifone	*C. tyrrhena*	80	73	30	80	35	43	10	15	5	15	2,5	13	5	4	2,5	2	4,8	1,4	2,6	4	0	2	14	1	2	0
14. Mt Grifone	*C. tyrrhena*	160	63	16	210	10	35	10	10	4	12	2	15	9	4,5	3	2	4,6	1,4	2,6	5	4	3	16	1	2	0
14. Mt Grifone	*C. tyrrhena*	520	110	30	60	30	40	18	15	10	20	4,5	16	14,5	3,5	2	2	4,8	1,6	2,6	5	7	4	13	1	2	0
14. Mt Grifone	*C. tyrrhena*	440	130	40	60	25	40	20	30	10	25	11	16	15	8	4	1,5	4,8	1,4	2,6	7	8	3	14	1	2	0
14. Mt Grifone	*C. tyrrhena*	600	140	45	85	40	35	18	25	10	20	2	15	16	7	3	2	4,8	1,6	2,6	5	6	5	14	1	2	0
14. Mt Grifone	*C. tyrrhena*	545	130	42	60	20	25	10	25	10	20	15	15	13	8	3	1,5	4,6	1,4	2,6	5	5	5	15	1	2	0
14. Mt Grifone	*C. tyrrhena*	550	67	20	75	35	42	18	20	3	15	2	15	14	7	4	1	4,8	1,6	2,6	6	4	9	22	1	2	0
14. Mt Grifone	*C. tyrrhena*	610	130	40	50	25	35	19	20	10	20	11	16	15	8	4	1,5	4,6	1,4	2,6	5	3	6	18	1	2	0
14. Mt Grifone	*C. tyrrhena*	370	145	42	70	25	40	22	20	10	20	10	11	11	4	2	1,5	4,8	1,4	2,6	5	5	4	15	1	2	0
14. Mt Grifone	*C. tyrrhena*	610	140	50	55	45	85	32	20	15	25	11	16	16	8	3	1,5	4,8	1,4	2,6	5	4	10	18	1	2	0
14. Mt Grifone	*C. tyrrhena*	865	145	45	90	50	25	10	20	15	20	13	15	16	6	3	2	4,8	1,4	2,6	5	7	12	19	1	2	0
14. Mt Grifone	*C. tyrrhena*	545	140	38	50	30	45	20	20	15	20	10	12	13	5	2	1	4	2	2,5	5	3	6	10	1	2	0
14. Mt Grifone	*C. tyrrhena*	435	135	36	55	30	45	20	20	15	20	15,5	15	16	5	3	1,5	4,4	2	2,5	5	5	4	15	1	2	0
15. Mongerbino	*C. todaroi*	640	300	90	83	80	85	20	25	5	60	14	14	14	6,5	4	1	4	1,4	2	5	9	20	16	0	2	1
15. Mongerbino	*C. todaroi*	640	260	65	85	55	100	15	25	5	60	5	15	12	7	2	1,8	4	1,4	2,1	5	4	12	15	0	2	1
15. Mongerbino	*C. todaroi*	550	320	120	40	22	100	36	25	5	75	6	15	12	6	3	1,5	4	1,5	2	7	4	16	20	0	2	0
15. Mongerbino	*C. todaroi*	360	130	70	23	14	85	20	20	6	50	5	13,5	11,5	7	4	1,5	4,1	1,4	2	7	4	2	15	0	2	1
15. Mongerbino	*C. todaroi*	390	150	80	25	10	75	20	30	20	50	6	14	14,5	6	3	2	4	1,4	2,1	7	4	4	15	0	2	1
15. Mongerbino	*C. todaroi*	670	180	90	120	70	85	20	25	20	60	10	14	10,5	5	4	2	4,1	1,4	2	7	5	16	18	0	2	1
15. Mongerbino	*C. todaroi*	670	175	76	60	35	80	15	25	20	60	13,5	12	15	8	4	2	4	1,5	2	7	5	15	20	0	2	1
15. Mongerbino	*C. todaroi*	460	150	60	45	20	78	16	25	20	60	14	13	14	6	4	2	4	1,4	2,1	7	4	16	12	0	2	0
15. Mongerbino	*C. todaroi*	670	180	85	60	50	85	20	25	20	60	10	12	13,5	5	4	2	4,1	1,4	2	7	4	16	18	0	2	1
15. Mongerbino	*C. todaroi*	500	180	90	50	25	78	16	25	20	60	11,5	12	15	4	3	1	4	1,4	2	7	4	12	12	0	2	0
16. Mt Catalfano	*C. todaroi*	270	145	30	50	25	90	25	25	4	25	5,5	15	14	4	3	1	5	2	2	5	4	1	19	1	2	1
16. Mt Catalfano	*C. todaroi*	960	225	55	75	50	55	15	30	3	40	17	14	13	6	3,5	2	4,8	2	2,1	8	5	4	11	0	2	1
16. Mt Catalfano	*C. todaroi*	740	220	50	85	45	60	20	30	4	40	9	15	13	5	3	1	4,8	2	2	8	9	7	15	0	2	0
16. Mt Catalfano	*C. todaroi*	510	225	55	80	30	60	20	30	4	35	14,5	14	14	5	3	1,5	4,7	1,9	2	8	8	4	18	0	3	0
16. Mt Catalfano	*C. todaroi*	530	145	35	80	30	55	18	28	4	35	21	12,5	17	3	2	2	3,8	1,6	2,2	8	8	12	9	0	3	0
16. Mt Catalfano	*C. todaroi*	330	145	33	65	25	50	15	28	4	35	12	16	20	4	3	2	3,8	1,6	2,4	7	3	2	11	0	1	0
16. Mt Catalfano	*C. todaroi*	440	125	50	60	25	55	19	28	5	30	20	15	13	5	3	2	3,8	1,3	2,8	7	4	2	14	1	1	0
16. Mt Catalfano	*C. todaroi*	385	145	35	35	25	95	15	30	5	20	10	16	14	5	3	2,5	4	1,6	2	5	2	18	19	0	1	0
16. Mt Catalfano	*C. todaroi*	400	145	30	45	20	105	15	30	5	20	13	14	14	6	4	2	4	1,6	2,1	5	3	9	20	0	1	0
16. Mt Catalfano	*C. todaroi*	375	155	30	85	70	75	10	30	5	20	8	17,5	14,5	7	4	2	3,9	1,6	2	5	8	2	18	0	1	0
17. Tindari	*C. seguenzae*	490	145	35	70	50	65	7	20	5	25	20	12	11,5	7	4	2	3,1	1,4	2,8	4	4	2	10	0	1	0
17. Tindari	*C. seguenzae*	345	310	75	70	30	180	55	50	15	55	20	12	16	4	2	1,5	3,1	1,3	2,8	7	5	1	10	0	1	0
17. Tindari	*C. seguenzae*	205	195	70	70	30	80	20	30	18	25	11,5	10	10	5	3	1	3,1	1,3	2,7	5	5	1	8	0	1	0
17. Tindari	*C. seguenzae*	510	210	55	70	28	55	10	25	10	15	5	13	12,5	4	3	1	3,2	1,3	2,8	6	6	5	12	0	2	0
17. Tindari	*C. seguenzae*	330	160	40	65	30	96	40	17	10	18	8	12	14	5	3	2	3,1	1,4	2,8	5	5	13	12	1	2	0
17. Tindari	*C. seguenzae*	420	160	55	60	20	90	40	30	8	20	11	13	11	4	3	1	3,2	1,3	2,7	5	6	2	11	1	2	0
17. Tindari	*C. seguenzae*	345	300	72	71	32	185	55	54	17	58	22	12	16	4	2	1,5	3,3	1,4	2,8	7	5	2	9	0	1	0
17. Tindari	*C. seguenzae*	200	190	75	68	27	82	21	35	18	22	10	10	10	5	3	1	3,2	1,3	2,7	5	5	1	8	0	1	0
17. Tindari	*C. seguenzae*	220	187	70	76	31	78	25	45	22	25	9,5	12	12	5	3	1	3,2	1,3	2,8	5	5	1	9	0	1	0
17. Tindari	*C. seguenzae*	330	160	30	65	30	60	10	25	10	15	12	13	12	5	3	1	3,1	1,3	2,8	6	6	6	12	1	2	0
18. Tardara	*C. saccensis*	610	138	46	65	40	90	40	14	11	25	13	12	12	5	3	2	4,5	2	5	4	0	14	13	1	3	1
18. Tardara	*C. saccensis*	520	135	38	70	40	100	40	22	10	25	9	15	14	6	3	2	4,5	1,9	5	5	5	8	15	0	3	1
18. Tardara	*C. saccensis*	535	130	35	80	35	90	45	24	14	20	4	18	13	4	3	2	4,6	2	4,8	6	4	3	14	0	3	1
18. Tardara	*C. saccensis*	390	125	30	65	20	90	40	20	14	25	5,5	14	12	5	3	1,8	4,8	1,9	4,8	6	4	1	14	0	3	1
18. Tardara	*C. saccensis*	430	140	35	35	25	95	40	26	12	20	5	11	12	6	3	2,5	4,5	1,9	5	3	3	3	13	1	3	1
18. Tardara	*C. saccensis*	550	125	32	50	25	95	40	20	10	25	13	14	13	6	3	2,5	4,6	2	5	6	3	8	13	0	3	1
18. Tardara	*C. saccensis*	530	120	30	90	58	95	40	24	14	20	12	12	14	5	4	2	4,7	2	4,8	5	3	6	13	0	3	1
18. Tardara	*C. saccensis*	420	130	35	70	22	85	40	22	12	25	5,5	14	12	5	3	1,8	4,8	1,9	4,8	6	4	4	14	0	3	1
18. Tardara	*C. saccensis*	550	140	44	55	40	80	35	17	12	25	11	12	12	5	3	1,9	4,5	2	4.9	4	0	12	13	1	3	1
18. Tardara	*C. saccensis*	500	130	40	48	32	88	40	20	10	22	12	14	13	6	3	2,2	4,2	2	4,7	6	2	9	12	0	3	1
