# Supplementary material for: A new species within the Centaureabusambarensis complex (Asteraceae, Cardueae) from Sicily
Source: Biodivers Data J. 2022 Oct 6;10:e91505. doi: 10.3897/BDJ.10.e91505 (PMC9836586; doi:10.3897/BDJ.10.e91505)
Supplement: Supplementary material 2 — Plots of the 19 continuous numeric characters [file bdj-10-e91505-s002.pdf]

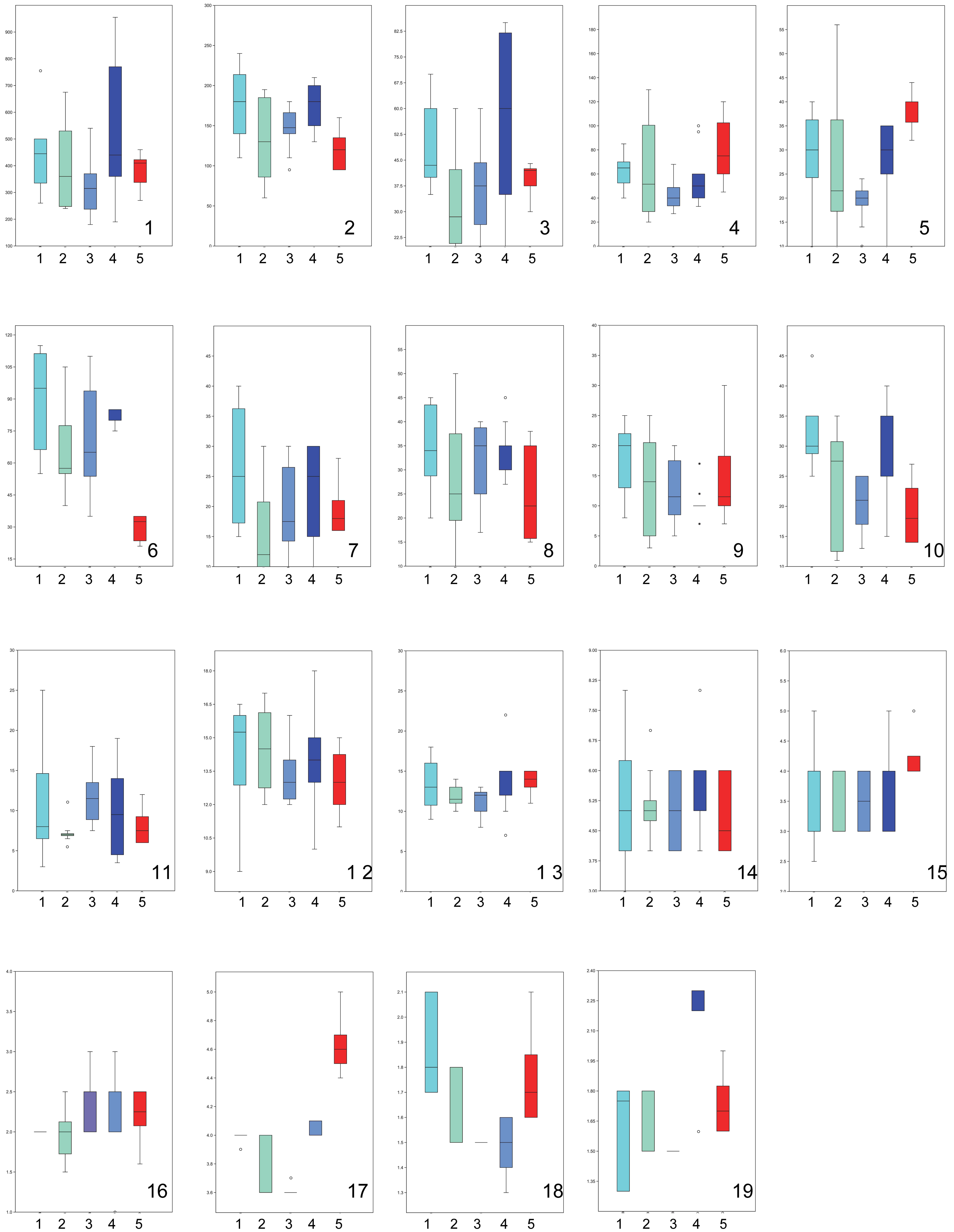

Suppl. Material 2. Plots of the 19 continuous numeric characters. For each sample, the 25–75% quartiles are drawn using a box. The median is shown with a horizontal line inside the box. The whiskers are drawn from the top of the box up to the largest data point less than 1.5 times the box height from the box, and similarly below the box. Outlier values are shown as dots. All measures in mm. The characters and the populations are numbered as in the text.
