# Supplementary material for: A new species within the Centaureabusambarensis complex (Asteraceae, Cardueae) from Sicily
Source: Biodivers Data J. 2022 Oct 6;10:e91505. doi: 10.3897/BDJ.10.e91505 (PMC9836586; doi:10.3897/BDJ.10.e91505)
Supplement: Supplementary material 3 — Confusion matrices of the Discriminant Analyses [file bdj-10-e91505-s003.pdf]

Suppl. Material 3. Confusion matrices of the Discriminant Analyses.

3.1. Confusion matrix of the Discriminant Analysis done on the 18 populations of the *C. busambarensis* group.

	C. busambarensis	cfr. *C. busambarensis*	*C. erycina*	*C. panormitana*	*C. tyrrhena*	*C. aegusae*	*C. todaroi*	*C. seguenzae*	*C. saccensis*	Total
C. busambarensis	43	0	0	0	0	0	0	0	0	43
cfr. *C. busambarensis*	0	10	0	0	0	0	0	0	0	10
C. erycina	0	0	10	0	0	0	0	0	0	10
C. panormitana	0	0	1	36	0	0	0	0	0	37
C. tyrrhena	0	0	0	1	37	0	0	0	0	38
C. aegusae	0	0	0	0	0	10	0	0	0	10
C. todaroi	0	0	0	0	1	0	19	0	0	20
C. seguenzae	0	0	0	0	0	0	0	10	0	10
C. saccensis	0	0	0	0	0	0	0	0	10	10
Total	43	10	11	37	38	10	19	10	10	188

3.2. Confusion matrix of the Discriminant Analysis with the Jackknife method done on the 18 populations of the *C. busambarensis* group.

	C. busambarensis	cfr. *C. busambarensis*	*C. erycina*	*C. panormitana*	*C. tyrrhena*	*C. aegusae*	*C. todaroi*	*C. seguenzae*	*C. saccensis*	Total
C. busambarensis	42	0	0	0	1	0	0	0	0	43
cfr. *C. busambarensis*	0	10	0	0	0	0	0	0	0	10
C. erycina	0	0	10	0	0	0	0	0	0	10
C. panormitana	0	0	3	30	4	0	0	0	0	37
C. tyrrhena	0	0	0	3	34	0	1	0	0	38
C. aegusae	0	0	0	0	0	10	0	0	0	10
C. todaroi	1	0	0	0	3	0	16	0	0	20
C. seguenzae	0	0	0	0	0	0	0	10	0	10
C. saccensis	0	0	0	0	0	0	0	0	10	10
Total	43	10	13	33	42	10	17	10	10	188

3.3. Confusion matrix of the Discriminant Analysis done on the 4 populations of *C. busambarensis* and the population from Rocche del Crasto.

	1. Busambra	2. Kumeta	3. Pizzuta	4. Isnello	5. Crasto	Total
1. Busambra	9	1	0	0	0	10
2. Kumeta	1	9	0	0	0	10
3. Pizzuta	0	0	12	0	0	12
4. Isnello	0	0	0	11	0	11
5. Crasto	0	0	0	0	10	10
Total	10	10	12	11	10	53

3.4. Confusion matrix of the Discriminant Analysis with the Jackknife method done on the 4 populations of *C. busambarensis* and the population from Rocche del Crasto.

	1. Busambra	2. Kumeta	3. Pizzuta	4. Isnello	5. Crasto	Total
1. Busambra	6	3	1	0	0	10
2. Kumeta	4	1	5	0	0	10
3. Pizzuta	0	2	10	0	0	12
4. Isnello	0	1	1	9	0	11
5. Crasto	0	0	0	0	10	10
Total	10	7	17	9	10	53
